# Predictive Models for the Transition from Mild Neurocognitive Disorder to Major Neurocognitive Disorder: Insights from Clinical, Demographic, and Neuropsychological Data

**DOI:** 10.3390/biomedicines12061232

**Published:** 2024-06-01

**Authors:** Anna Tsiakiri, Christos Bakirtzis, Spyridon Plakias, Pinelopi Vlotinou, Konstantinos Vadikolias, Aikaterini Terzoudi, Foteini Christidi

**Affiliations:** 1 Neurology Department, School of Medicine, Democritus University of Thrace, 68100 Alexandroupolis, Greece; atsiakir@med.duth.gr (A.T.); kvadikol@med.duth.gr (K.V.); terzoudi@med.duth.gr (A.T.); 2 B’ Department of Neurology and the MS Center, School of Medicine, AHEPA University Hospital, Aristotle University of Thessaloniki, 54124 Thessaloniki, Greece; cbakirtzis@auth.gr; 3 Department of Physical Education and Sport Science, University of Thessaly, 41500 Trikala, Greece; spyros_plakias@yahoo.gr; 4 Department of Occupational Therapy, University of West Attica, 12243 Athens, Greece; pvlotinou@uniwa.gr

**Keywords:** mild cognitive impairment, neurocognitive disorders, prediction, neuropsychological tests, risk factors, body mass index, alcohol drinking, depression, demographic factors, longitudinal studies

## Abstract

Neurocognitive disorders (NCDs) are progressive conditions that severely impact cognitive function and daily living. Understanding the transition from mild to major NCD is crucial for personalized early intervention and effective management. Predictive models incorporating demographic variables, clinical data, and scores on neuropsychological and emotional tests can significantly enhance early detection and intervention strategies in primary healthcare settings. We aimed to develop and validate predictive models for the progression from mild NCD to major NCD using demographic, clinical, and neuropsychological data from 132 participants over a two-year period. Generalized Estimating Equations were employed for data analysis. Our final model achieved an accuracy of 83.7%. A higher body mass index and alcohol drinking increased the risk of progression from mild NCD to major NCD, while female sex, higher praxis abilities, and a higher score on the Geriatric Depression Scale reduced the risk. Here, we show that integrating multiple factors—ones that can be easily examined in clinical settings—into predictive models can improve early diagnosis of major NCD. This approach could facilitate timely interventions, potentially mitigating the progression of cognitive decline and improving patient outcomes in primary healthcare settings. Further research should focus on validating these models across diverse populations and exploring their implementation in various clinical contexts.

## 1. Introduction

Neurocognitive disorders (NCDs) encompass a wide range of conditions characterized by a decline in cognitive functioning, with Alzheimer’s disease being the most well-known. According to the Diagnostic and Statistical Manual of Mental Disorders, fifth edition (DSM-5), mild neurocognitive disorder (mild NCD) is defined as a noticeable decline in cognitive functioning that does not significantly interfere with daily activities [1,2]. This includes conditions like mild cognitive impairment (MCI), which serves as a precursor to major neurocognitive disorder (major NCD) [3,4,5]. Major NCD is characterized by a significant decline in cognitive abilities that impairs daily life, affecting memory, language, and other cognitive functions [6]. Major NCD, previously known as dementia, is defined by the Diagnostic and Statistical Manual of Mental Disorders, fifth edition (DSM-5), as a significant cognitive decline from a previous level of performance in one or more cognitive domains (complex attention, executive function, learning and memory, language, perceptual–motor function, and social cognition). This decline must be substantial enough to interfere with independence in daily activities [7]. Major NCD can arise from various etiologies, including Alzheimer’s disease, vascular disease, traumatic brain injury, and other conditions. Symptoms vary depending on the cause but generally include memory loss, impaired judgment, language difficulties, and changes in personality and behavior [8]. The diagnostic process for major NCD involves a thorough clinical assessment, including medical history, cognitive testing, and possibly neuroimaging or laboratory tests to identify the underlying cause and rule out other conditions. Clinicians use standardized tools like the Mini-Mental State Examination (MMSE) or the Montreal Cognitive Assessment (MoCA) to evaluate cognitive decline [9].

Neurocognitive disorders represent a significant and growing public health concern as populations age. Alzheimer’s disease, the most prevalent form of major NCD, affects millions worldwide and incurs substantial emotional and economic costs [10]. These disorders not only affect the individuals diagnosed but also have profound impacts on families and caregivers, often necessitating long-term care and support. Understanding the distinctions between mild NCD and major NCD is crucial for early intervention and management [11]. While mild NCD might not severely disrupt daily life, it signals a higher risk of progressing to more severe forms, highlighting the need for early detection and therapeutic strategies.

The intermediate phase between minor NCD and major NCD is characterized by mild cognitive impairments that, while noticeable, do not severely impact daily activities. Common symptoms include memory loss, difficulty with problem solving, challenges with planning and organizing, and language difficulties, as well as decreased attention span and concentration [12]. Patients in this stage often retain independence but may need help with complex tasks and may experience frustration or anxiety due to their cognitive challenges, with changes sometimes first noticed by friends and family [1]. Studies on minor NCD often include patients in this intermediate phase to track progression to major NCD, highlighting the importance of longitudinal research to monitor cognitive, structural, and biomarker changes over time [2]. Progression from minor to major NCD varies widely, typically occurring within 3 to 5 years, and is influenced by factors such as age, genetics, and comorbid conditions [13].

Early detection and intervention in neurocognitive disorders are paramount for improving patient outcomes. Predictive models that can accurately identify individuals at risk for progressing from mild NCD to major NCD are crucial for implementing timely interventions that can slow cognitive decline [14]. Recent advancements have demonstrated the transformative impact of artificial intelligence (AI)-based techniques in early detection and diagnosis, emphasizing the potential of these tools in clinical settings [15,16]. AI approaches have been applied to detect early signs of cognitive impairment, allowing for more timely and precise diagnoses. This is essential as the aging population grows and the prevalence of dementia increases globally.

Timely intervention can significantly alter the trajectory of neurocognitive disorders. For instance, lifestyle modifications, cognitive training, and pharmacological treatments can be more effective if initiated during the early stages of cognitive decline. AI and machine learning models are particularly promising in this regard. By analyzing vast datasets, these technologies can identify subtle patterns and predictors of disease progression that might be missed by traditional diagnostic methods. Moreover, AI can enhance the accuracy and efficiency of neuropsychological assessments, reducing the burden on healthcare systems and providing more accessible diagnostic options for patients.

Several studies have focused on identifying predictive markers for the transition from mild NCD to major NCD. Cognitive performance, particularly episodic memory deficits, has consistently been highlighted as a robust predictor of progression. Delayed recall in episodic memory tests is one of the most significant indicators of future decline [17,18,19]. Additionally, biomarkers, such as cerebrospinal fluid (CSF) tau and amyloid levels, and neuroimaging findings, like hippocampal atrophy, have been integrated with cognitive assessments to enhance predictive accuracy [20,21,22].

Biomarkers play a critical role in the early detection of neurocognitive disorders. For example, AI-driven approaches have shown potential in improving predictive accuracy by analyzing complex patterns of brain connectivity. Another study highlighted the importance of transdiagnostic biomarkers, which can provide insights across different neurocognitive and psychiatric disorders, thereby improving the overall understanding of disease mechanisms [23]. Integration of multiomics profiles and multimodal electroencephalographic (EEG) data has contributed significantly to personalized diagnostic strategies, enhancing the precision of early detection methods [24].

The field of biomarker research is rapidly evolving, with significant advancements in both molecular and imaging techniques. CSF biomarkers, such as tau protein and amyloid-beta, have been extensively studied for their roles in the pathophysiology of Alzheimer’s disease [21]. Elevated levels of these proteins are associated with neuronal damage and plaque formation, key features of Alzheimer’s pathology. Additionally, neuroimaging techniques, including magnetic resonance imaging (MRI) and positron emission tomography (PET) scans, provide critical insights into brain structure and function, enabling the identification of atrophy patterns and metabolic changes associated with cognitive decline [25].

Functional biomarkers, such as changes in brain connectivity observed through resting-state functional MRI, have also shown promise in early detection. These biomarkers can reveal disruptions in neural networks that precede clinical symptoms, offering a window for early intervention [26]. Moreover, advancements in genomics and proteomics are paving the way for the discovery of novel biomarkers that could further refine diagnostic accuracy and prognostic assessments [27].

Genetic factors also play a crucial role in predicting cognitive decline. The presence of the apolipoprotein E (APOE) ε4 allele has been strongly associated with an increased risk of Alzheimer’s disease and faster cognitive decline [18]. Demographic factors, including age, education, and gender, further influence the risk of progression from mild NCD to major NCD. For instance, older age and lower educational attainment are associated with higher risks [21,22]. The interplay between genetics and environmental factors is complex and multifaceted [28,29,30]. While the APOE ε4 allele is the most well-established genetic risk factor for Alzheimer’s disease, other genes are also being investigated for their roles in neurocognitive disorders [31]. Genome-wide association studies (GWASs) have identified numerous genetic variants that contribute to disease susceptibility, highlighting the polygenic nature of these conditions [32]. Understanding the genetic architecture of neurocognitive disorders can inform personalized medicine approaches, where interventions are tailored based on an individual’s genetic profile [33].

Demographic factors also offer valuable insights into disease risk and progression. Studies have shown that individuals with higher educational attainment or greater cognitive reserve tend to exhibit a slower rate of cognitive decline [34]. This suggests that lifelong cognitive engagement may confer protective effects against neurocognitive disorders. Additionally, sex differences in disease prevalence and progression rates have been observed, with women generally exhibiting a higher risk of developing Alzheimer’s disease [35]. Hormonal factors, lifestyle differences, and genetic variations may all contribute to these disparities [36].

Despite advancements in identifying individual risk factors, comprehensive models that integrate multiple data sources to predict progression to major NCD are still lacking. Most studies have examined cognitive markers, genetic factors, and biomarkers separately, but there is a need for a unified approach that combines these elements to enhance predictive capabilities. Furthermore, longitudinal studies are needed to better understand the trajectory of cognitive decline and biomarker changes in individuals with mild NCD [37]. Recent findings underscore the urgent need for innovative and cost-effective early-stage intervention strategies to address the growing global challenge of dementia [38].

The development of comprehensive predictive models involves integrating diverse datasets, including clinical, genetic, biomarker, and neuroimaging data [39]. Machine learning and artificial intelligence are instrumental in this endeavor, offering powerful tools to analyze complex datasets and generate predictive models with high accuracy [40]. These models can identify individuals at high risk for progression to major NCD, facilitating early intervention and personalized treatment plans. Longitudinal studies are particularly valuable in understanding the natural history of neurocognitive disorders. By following individuals over time, researchers can observe the progression of cognitive decline and identify early markers of disease. Such studies also allow for the assessment of intervention efficacy, providing critical insights into which treatments are most effective at different stages of the disease [41].

In conclusion, the integration of AI-based techniques, biomarkers, genetic factors, and comprehensive predictive models holds significant promise for the early detection and intervention of neurocognitive disorders. As the prevalence of dementia continues to rise globally, these advancements are crucial for enhancing diagnostic accuracy, understanding disease mechanisms, and developing personalized treatment strategies. Ongoing research and longitudinal studies are essential to refine these approaches and ensure their effective implementation in clinical settings. By leveraging the power of technology and multidisciplinary research, we can make significant strides in combating the challenges posed by neurocognitive disorders and improving the quality of life of affected individuals.

The current study aims to develop and validate predictive tools that enhance the screening and risk assessment of major NCD in primary healthcare settings by integrating clinical, demographic, and neuropsychological data.

## 2. Materials and Methods

A brief description of the study’s methodological approach towards the inclusion of predictive factors and the implementation of specific models for data analysis follows. The methodology of this study was carefully designed to ensure robust and reliable data collection, analysis, and interpretation. The study aimed to develop and validate predictive models for the transition from minor neurocognitive disorder (minor NCD) to major neurocognitive disorder (major NCD). We employed a comprehensive approach that integrates demographic, clinical, and neuropsychological assessments to identify significant predictors of cognitive decline. The study design and methodology are grounded in established research practices and supported by the relevant literature to enhance the validity of our findings. Prior studies have demonstrated the importance of longitudinal data in understanding the progression of neurocognitive disorders [12,13]. Additionally, the use of neuropsychological tests, such as the CAMCOG and the GDS, has been validated in various settings to predict cognitive decline [42,43]. Our approach ensures a comprehensive evaluation of factors contributing to the transition from minor to major NCD, providing a robust framework for early intervention strategies.

Furthermore, the international literature emphasizes the importance of focusing on the structure of the data and correlations in Generalized Estimating Equations (GEE) analysis rather than a fixed minimum number of observations per variable [44,45,46]. GEE analysis can be applied even to small sample sizes [47]. However, it is worth noting that, in our research, we adhered to the general assumption for regression models that requires a minimum threshold of 10 observations per independent variable [48,49,50]. In our case, there were 394 observations/24 variables = 16.4 observations per variable.

### 2.1. Subjects

**Figure 1:**
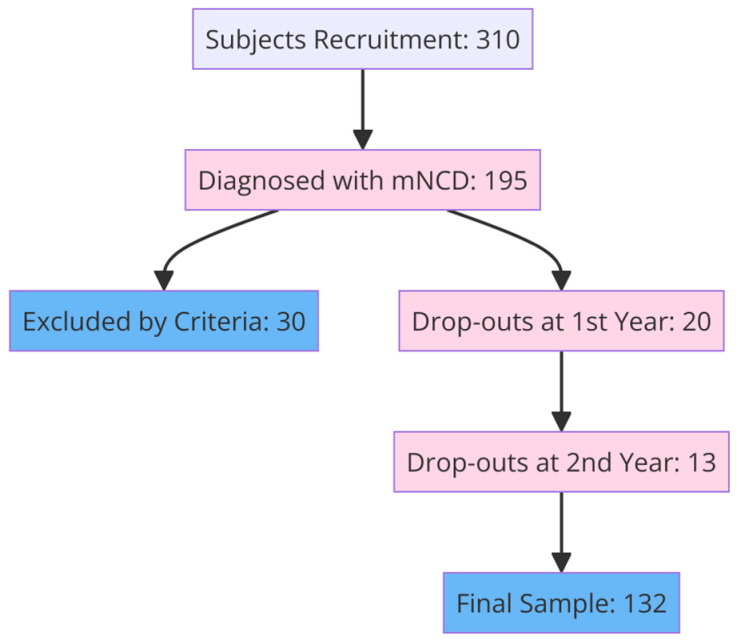
Subjects’ recruitment process.

Data regarding 132 participants in an ongoing registry of the Neurology Department of the University Hospital of Alexandroupolis with minor NCD and with available diagnostic follow-up assessments for at least 2 years were included in the study. The final sample consisted of participants who visited the outpatient dementia clinic and underwent the examination as a part of their routine neuropsychological assessment (Figure 1).

All participants signed an informed consent form prior to their participation. Approval was also required for the patients with dementia by their caregiver and/or by a legal representative. Approval for the study was granted by the Ethics Committee of the University Hospital of Alexandroupolis (ΔΣ1/Θ68/06-04-2020). The data were analyzed anonymously.

### 2.2. Inclusion/Exclusion Criteria

The interview collected biographical information and medical data, including information regarding any medical diagnosis; history of cardiovascular, metabolic, and neurological syndromes; and history of affective diseases. Individuals with NCDs underwent a neurological examination, neuropsychological assessment, neuroimaging, and specific biochemical and hematological testing.

A total of 132 individuals at baseline met the diagnostic criteria for minor NCD as defined in the fifth edition of the Diagnostic and Statistical Manual of Mental Disorders (DSM-V) [51]. These criteria include the following: (a) self-reported or observed decline in cognitive functioning by the patient, family member, or clinician; (b) cognitive impairment for the individual’s age demonstrated by formal neuropsychological testing; (c) evidence of gradual cognitive decline in objective tasks beyond normal aging but not meeting criteria for dementia; (d) preserved general cognitive and daily function; and (e) no prior diagnosis of dementia or other conditions (e.g., depression, delirium, intoxication, or psychosis) that could explain the impairment. Additional inclusion criteria were as follows: age over 40 years; mild cognitive decline based on Mini-Mental State Examination (MMSE) [52,53] and Montreal Cognitive Assessment (MoCA) score [54,55], defined as 1 to 1.5 standard deviations (SDs) below the mean for age- and education-adjusted norms based on normative data; absence of other neurological diseases; not currently taking cholinesterase inhibitors, antipsychotics, and/or anticholinergic drugs. The inclusion criterion of age over 40 years in studies of minor NCD is significant for capturing early markers of cognitive decline and understanding the progression of the disorder. While cognitive decline is more common in older adults, including patients starting at age 40 helps identify early symptoms of minor NCD, as this age group may begin to show subtle signs of cognitive impairment [12]. This inclusion is associated with several critical parameters: genetic factors like the presence of the APOE ε4 allele may start influencing cognitive decline in individuals in their 40s [2]; lifestyle factors, such as diet, exercise, and smoking, as well as comorbidities like hypertension, diabetes, and cardiovascular diseases, begin to impact cognitive health in midlife, increasing the risk of minor NCD [13]; and neuropsychological changes in cognitive performance and brain health can show measurable changes in this age group, making it a critical period for early detection and intervention [1]. Therefore, including patients over 40 years old provides valuable insights into the early onset and progression of cognitive decline.

The exclusion criteria were as follows: secondary causes of cognitive deficits confirmed with laboratory tests, including vitamin B12/folate determination and thyroid functioning tests; structural lesions on conventional brain MRI, such as territorial infarction, intracranial hemorrhage, brain tumor, hydrocephalus, and traumatic brain injury. The subjects were followed annually with two repeated clinical visits after baseline.

### 2.3. Conversion to Major NCD

Our primary study outcome was progression to major NCD. In order to avoid circularity, the major NCD diagnostic criteria did not include any of our predictive neuropsychological testing markers. We used the criteria based on modes of assessment that were independent of our neuropsychological decline measure. Participants were diagnosed with major NCD [56] based on DSM-V and decline in activities of daily living as well as cognitive test scores for MMSE and MoCa (total scores) falling 2 or more standard deviations (SDs) below the mean based on available normative data.

### 2.4. Neuropsychological Predictive Factors

All participants underwent a battery of neuropsychological tests comprising the Greek version of the Cambridge Cognitive Examination Scale (CAMCOG) as part of the Cambridge Mental Disorders of the Elderly (CAMDEX) [42,57]. The cognitive domains assessed through the subtasks were abilities of praxis, orientation, understanding, language, memory, and perception. Confrontation naming was evaluated by the Boston Naming Test (BNT) [58] and executive functioning by the Functional Cognitive Assessment Scale (FUCAS) [59]. The Functional Rating Scale of Symptoms of Dementia (FRSSD) was administered to assess the patient’s functionality in daily activities based on the caregiver’s perspective. Emotional status was evaluated by the Geriatric Depression Scale (GDS) for the detection of depressive symptoms [43,60]. The Hamilton Depression Scale (HAM-D) [61] was administered to assess patients’ emotional states through 17 questions from the caregiver’s perspective. Patients’ neuropsychiatric disturbances were assessed through the Neuropsychiatric Inventory (NPI) administered to caregivers [62]. The neuropsychological examinations took place in a quiet room, and every participant was tested individually by the same neuropsychologist of the Neurology Department.

### 2.5. Demographic and Clinical Predictive Factors

Age (years), sex (male/female), and education (years) were the demographic factors studied, while the clinical variables included duration of minor NCD (years), body mass index (BMI) ≥ 25 [63] (yes/no), smoking (yes/no), alcohol consumption (yes/no), history of cardiovascular disease (yes/no), presence of white matter lesions (yes/no), and cerebrovascular burden (at least one of the following risk factors: atrial fibrillation, cerebrovascular diseases, hypertension, diabetes, and hypercholesterolemia).

### 2.6. Statistical Analysis

Data were analyzed using a Generalized Estimating Equations (GEE) framework to account for the longitudinal nature of the data with repeated measures over time (baseline, first year, and second year) [64,65]. The dependent variable was diagnosis, coded as 0 for minor NCD and 1 for major NCD. The independent variables included in the model are shown in Table 1 and Table 2. The model was specified with a binomial distribution and a logit link function. The working correlation structure was set as unstructured, allowing for a general form of the covariance matrix for the repeated measures [66,67]. Employing an unstructured correlation matrix is beneficial when the intervals between measurements are uniform across subjects [44], which aligns with the methodology of our study. Wald χ^2^ tests were used to determine the significance of the predictors, with a 95% confidence interval. All analyses and graphical representations were performed using SPSS (version 25.00) software, and the significance level was set at *p* < 0.05.

## 3. Results

The Quasi-Likelihood under Independence Model Criterion (QIC) was 428.865, and the Corrected Quasi-Likelihood under Independence Model Criterion (QICC) was 454.655, indicating the model’s adequacy in fitting the data in comparison to alternative models that were tested. The model performed well, with an accuracy of 83.7%. Table 1 shows the descriptive statistics for the categorical (nominal/ordinal) and continuous (scale) variables of the model.

The GEE analysis revealed significant effects of sex, BMI ≥ 25, alcohol, CAMCOG-praxis, and GDS on the likelihood of major NCD (Table 2). Specifically, females were less likely to be diagnosed with major NCD than males (B = −0.664, OR = 0.515, Wald χ^2^(1) = 8.329, *p* = 0.004). Participants with increased BMI (BMI ≥ 25) had higher odds of receiving a major NCD diagnosis (B = 0.820, OR = 2.272, Wald χ^2^(1) = 9.069, *p* = 0.003). Similarly, alcohol consumption was associated with a higher likelihood of major NCD diagnosis (B = 0.857, OR = 2.356, Wald χ^2^(1) = 9.035, *p* = 0.003). Additionally, the results showed that higher scores on the CAMCOG—praxis were significantly associated with lower odds of being diagnosed with major NCD (B = −1.115, OR = 0.891, Wald χ^2^(1) = 6.396, *p* = 0.011). Furthermore, higher scores on the GDS were significantly associated with decreased odds of major NCD diagnosis (B = −1.137, OR = 0.872, Wald χ^2^(1) = 4.801, *p* = 0.028).

**Figure 2:**
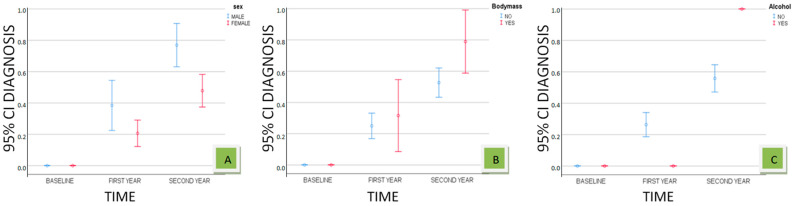
Estimated probabilities of major NCD diagnosis over time with 95% confidence intervals of means by sex, body mass, and alcohol consumption.

The interaction effects of time with sex, BMI ≥ 25, and alcohol consumption were visually explored through estimated marginal means plots. The charts in Figure 2 show the estimated probabilities of major NCD diagnosis for various subgroups based on sex, BMI ≥ 25, and alcohol consumption. The diagrams present the mean values and the 95% confidence intervals for each subgroup at three time points. The charts reveal the following: (A) males have a higher likelihood of major NCD diagnosis compared to females at all time points, with the differences being more pronounced in the second year; (B) individuals with excessive BMI (BMI ≥ 25) appear to have a higher likelihood of major NCD diagnosis in the second year compared to those without excessive weight; (C) individuals who consume alcohol have a greater likelihood of major NCD diagnosis in the second year compared to those who do not consume alcohol.

**Figure 3:**
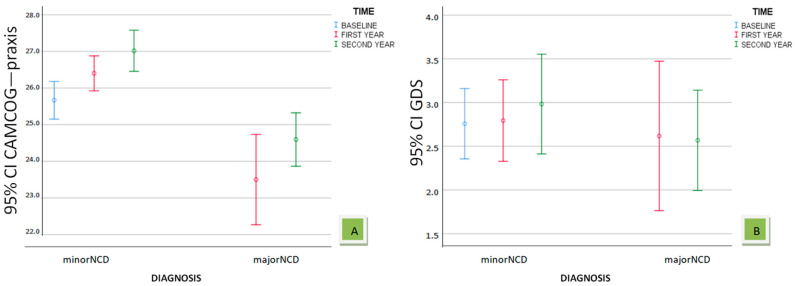
Changes in mean values of CAMCOG—praxis (**A**) and GDS (**B**) with 95% confidence intervals by diagnosis and time.

Figure 3 includes two charts with the 95% confidence intervals for the variables CAMCOG—praxis and GDS among patients with minor NCD and patients with major NCD over the time periods. Both in the first and second year, individuals displaying major NCD showed lower values in CAMCOG—praxis and GDS. Indeed, even visually, it appears that for CAMCOG—praxis the differences are statistically significant.

In summary, the results from the GEE show the influence of categorical variables like sex, BMI, and alcohol consumption on the likelihood of being diagnosed with major NCD. For instance, females showed a reduced likelihood of a major NCD diagnosis compared to males, those with a BMI over 25 were more likely to receive a major NCD diagnosis, and alcohol consumers also showed a higher probability of a major NCD diagnosis. Additionally, higher scores on the CAMCOG—praxis and higher scores on the GDS were associated with a decreased likelihood of a major NCD diagnosis.

## 4. Discussion

This study aimed to address the critical need for early detection and effective management of neurocognitive disorders by developing a validated predictive model for the transition from minor neurocognitive disorder (minor NCD) to major neurocognitive disorder (major NCD). By focusing on demographic, clinical, and neuropsychological factors, we aim to enhance early intervention strategies within primary healthcare settings, ultimately improving patient outcomes and quality of life. The ultimate goal of this research is to facilitate early identification of individuals at high risk for major NCD, enabling timely interventions that can delay or prevent severe cognitive decline. The primary challenge lies in integrating diverse data sources—demographic information, clinical history, and neuropsychological test results—into a cohesive and accurate predictive model. Overcoming this challenge requires extensive knowledge in neuropsychology, geriatrics, and advanced statistical modeling techniques.

Our study highlights a crucial predictive model for identifying individuals at risk of major NCD among those with minor NCD. Baseline clinical profiles, particularly emphasizing increased ΒΜΙ (BMI ≥ 25) and alcohol consumption, emerged as significant factors associated with heightened major NCD risk. Conversely, certain predictors appeared to lower the likelihood of major NCD, including being female, exhibiting higher scores on the GDS, and achieving higher scores in praxis assessments. Of note, to achieve the study’s goals, a deep understanding of neurocognitive disorders and the factors influencing their progression is essential. This includes expertise in neuropsychological assessment, familiarity with demographic and lifestyle risk factors, and proficiency in statistical methods for analyzing longitudinal data.

Alcohol consumption, defined as consuming more than one glass of alcohol per day based on self-reported questionnaires, emerged as a predictive factor for progression to major NCD. Considering previous research, Kuang et al. [68] highlighted alcohol consumption as a significant predictive factor in various models for dementia, emphasizing its importance in assessing cognitive health alongside other variables, such as age, activities of daily living questionnaire score, and smoking status. Conversely, Koch et al. [69] emphasized caution in alcohol consumption among individuals with minor NCD, consistent with Xu et al. [70] who identified a J-shaped relationship between alcohol intake and cognitive decline, suggesting that light to moderate drinking might reduce dementia risk in this vulnerable population. While moderate alcohol intake has been linked to potential cognitive benefits in certain contexts, such as reduced progression to major NCD in individuals with minor NCD [71], caution is warranted. Xu et al. [72] demonstrated a nonlinear association between alcohol consumption and dementia risk, suggesting that excessive drinking may actually elevate the risk.

The present study identified BMI (BMI ≥ 25) as a predictive factor for conversion to major NCD. This finding aligns with previous research exploring the complex relationship between BMI and cognitive decline. Hessler et al. [73] investigated cardiovascular health metrics and dementia risk in older adults. While their study did not find a direct association between BMI and dementia risk, it emphasized the importance of other cardiovascular risk factors, such as smoking, physical activity levels, and glucose levels, which could indirectly impact cognitive health. A more recent study [74] examined the effects of the MIND diet on cognition in older adults with a higher BMI and suggested that dietary interventions alone may not significantly alter cognitive outcomes, highlighting the complexity of factors influencing dementia risk beyond BMI. Mirza et al. [75] explored trajectories of depressive symptoms and their association with dementia risk. Although not directly related to BMI, their findings underscored the importance of mental health factors in cognitive decline, which could interact with BMI-related mechanisms. Another study [76] provided insights into the obesity paradox, where late-life lower BMI was associated with increased cortical amyloid burden and dementia risk, especially in APOE4 carriers.

In our comprehensive investigation into predictive factors associated with the conversion to major NCD, a notable finding emerged regarding the impact of female sex on dementia risk. Our analysis indicated that female sex is linked to a lower risk for major NCD progression, a conclusion that draws upon a body of research exploring sex-specific influences on cognitive health. Studies [77,78] have consistently observed a higher prevalence and elevated risk of AD among women compared to men. These findings suggest potential sex-specific vulnerabilities to certain forms of dementia, underscoring the need to explore biological and environmental factors that may contribute to these disparities. Conversely, other researchers [68,79] have highlighted educational disparities and hormonal fluctuations, particularly estrogen changes, as potential contributors to variations in dementia risk between the sexes. Moreover, recent studies [80,81] have reported differential associations of risk factors with dementia among men and women, indicating that certain risk factors may have distinct impacts based on sex.

Our research finding that increased scores on the GDS predict a slower progression to major NCD adds a significant dimension to the discourse on the potential of depressive symptoms as early indicators of cognitive impairment. This result is particularly insightful in the context of our study methodology, where participants identified with elevated GDS scores at baseline were subsequently enrolled in a comprehensive monitoring program. This intervention included both pharmacological treatment and counseling services for the participants and their family systems, aimed specifically at mitigating the impact of depressive factors on cognitive health. Supporting our approach, the study by Mirza et al. [75] aligns well with our findings, suggesting that increasing trajectories of depressive symptoms over time are associated with a higher risk of dementia, thereby reinforcing the notion that actively managing these symptoms could significantly alter the progression trajectory of cognitive decline. In contrast, another study [82] found that mild depressive symptoms did not predict AD, highlighting the importance of the severity and management of symptoms in their role as potential predictors. Moreover, while Nation et al. [83] emphasize the utility of neuropsychological markers in predicting dementia, the integration of therapeutic interventions in response to depressive symptomatology in our study provides a model for combining psychological and neuropsychological assessments to enhance predictive accuracy.

Our investigation aligns with previous studies, showing that improved praxis abilities can have a protective effect against cognitive decline. These abilities are integral to our daily cognitive functions and may play a role in mitigating the symptoms or delaying the progression of neurocognitive disorders. One study highlights the positive impact of literacy and education on visuo-constructional abilities, suggesting that educational activities enhancing these abilities could support cognitive health in the elderly [84]. This is particularly evident in individuals who engage in complex cognitive activities, showing better performance in cognitive tests compared to those with lower engagement levels. However, the relationship between praxis abilities and cognitive health is not straightforward. Martins-Rodrigues et al. found that certain visuo-constructional tasks did not effectively differentiate between individuals with minor NCD and healthy controls, suggesting that the protective effects of enhanced praxis abilities may be limited to specific cognitive functions or early stages of cognitive decline [85].

In summary, our results highlight the importance of specific demographic and lifestyle factors in predicting the transition from minor NCD to major NCD. Increased BMI and alcohol consumption were associated with a higher risk, while female sex, higher GDS scores, and better praxis abilities were linked to a lower likelihood of progression. These findings underscore the need for comprehensive risk assessments in primary care settings to identify and support at-risk individuals effectively.

This research is significant as it addresses a growing public health concern: the rising prevalence of neurocognitive disorders among aging populations. Early detection of individuals at high risk for major NCD can lead to timely interventions, potentially delaying the onset of severe cognitive decline and reducing the overall burden on healthcare systems.

### 4.1. Strenghts and Limitations of the Study

One of the study’s primary strengths is the integration of multiple types of predictive data, including clinical risk factors and neuropsychological tests, which make it a useful tool for general practitioners in primary healthcare settings. Additionally, the study focused on developing and validating predictive tools that can be effectively implemented within community-based healthcare frameworks. These tools are designed to facilitate early intervention strategies and improve patient outcomes, making them particularly valuable in remote areas where they can aid general practitioners in referring patients to specialized centers. Moreover, the longitudinal study design, which included follow-ups, allowed for a more accurate assessment of disease progression from minor NCD to major NCD.

The study has certain limitations. The sample size of 132 participants from a single institution may limit the generalizability of the findings to broader populations or different geographic locations, potentially affecting its utility in diverse or remote settings. There is also a need for further validation studies across diverse populations to ensure the model’s generalizability and clinical applicability. Additionally, the study acknowledges gaps in the literature where comprehensive models integrating various data sources (cognitive, genetic, and biomarker data) are still lacking.

### 4.2. Potential Clinical Implications and Future Research

The clinical applications of our findings are substantial. The predictive model can aid in the early identification of patients at risk of major NCD, facilitating timely referrals to specialized care and the implementation of personalized intervention strategies. The findings from this study pave the way for several avenues of future research. First, further refinement of predictive models is necessary to enhance their accuracy and reliability. This can be achieved by incorporating additional biomarkers and genetic information alongside the current demographic, clinical, and neuropsychological factors. Advanced machine learning techniques could also be employed to analyze these complex datasets more effectively. Secondly, the impact of targeted interventions based on the predictive model should be explored. Moreover, it is crucial to validate the predictive model across diverse populations. Finally, the integration of the predictive model into primary healthcare systems requires careful consideration of its implementation and usability by general practitioners. Research should focus on developing user-friendly tools and training programs that facilitate the adoption of these predictive models in everyday clinical practice.

## 5. Conclusions

The study successfully developed and validated predictive tools that integrate clinical risk factors and neuropsychological tests tailored for implementation in primary healthcare settings. These tools enable effective screening and risk assessment for neurocognitive disorders (NCDs), thereby facilitating early intervention strategies which can significantly improve patient outcomes. The findings underscore the influence of demographic factors, such as gender, with females exhibiting a lower risk of major neurocognitive disorder (MNCD) progression compared to males, suggesting potential sex-specific interventions. Additionally, lifestyle factors like BMI and alcohol consumption were significant in MNCD progression, presenting opportunities for targeted interventions to delay or prevent severe cognitive impairments. Moreover, certain neuropsychological predictors appeared to lower the likelihood of major NCD, including exhibiting higher scores on the GDS and achieving higher scores in praxis assessments. The proposed comprehensive and validated model can be adaptable to various healthcare settings and is poised to significantly influence clinical practice, especially in primary care environments, by providing tools that support early diagnosis and tailored intervention strategies.

## Figures and Tables

**Table 1:** Descriptive statistics for baseline demographic, clinical, and cognitive data.

	N	Percent	Mean	Std.	Min.	Max.
Age (years)	132	-	70.6	7.5	44	83
Sex (M/F)	39/93	29.5/70.5				
Education (years)	132	-	8.3	4.8	0	18
Duration (years)	132	-	1.7	1.3	0	11
BMI ≥ 25 (Y/N)	20/112	15.2/84.8	-	-	-	-
WML (Y/N)	41/91	31.1/68.9	-	-	-	-
Cardiovascular diseases (Y/N)	34/98	25.8/74.2	-	-	-	-
Smoking (Y/N)	16/116	12.1/87.9	-	-	-	-
Alcohol (Y/N)	2/130	1.5/98.5	-	-	-	-
CB (3/2/1/0)	7/30/40/55	5.3/22.7/30.3/41.7	-	-	-	-
CAMCOG—praxis	132	-	25.7	3.0	13	30
CAMCOG—orientation	132	-	9.8	0.6	6	10
CAMCOG—understanding	132	-	6.3	0.8	4	7
CAMCOG—language	132	-	23.3	2.8	15	29
CAMCOG—memory	132	-	18.9	3.8	6	25
CAMCOG—perception	132	-	17.2	3.8	1	24
CAMCOG—time perception	132	-	1.9	1.2	0	14
BNT	132	-	47.0	7.3	29	60
BNT—time completion	132	-	489.5	159.6	170	875
FUCAS	132	-	45.9	8.8	42	88
GDS	132	-	2.8	2.3	0	9
HAM-D	132	-	4.6	3.3	0	21
FRSSD	132	-	4.3	2.9	0	14
NPI	132	-	2.1	2.7	0	14

Notes. M/F: Male/female; BMI: Body mass index; WML: White matter lesions; Y/N: Yes/no; CB: Cerebrovascular burden; CB-0: No cerebrovascular factor among atrial fibrillation, cerebrovascular diseases, hypertension, diabetes, and hypercholesterolemia; CB-1: One cerebrovascular factor among atrial fibrillation, cerebrovascular diseases, hypertension, diabetes, and hypercholesterolemia; CB-2: Two cerebrovascular factors among atrial fibrillation, cerebrovascular diseases, hypertension, diabetes, and hypercholesterolemia; CB-3: Three cerebrovascular factors among atrial fibrillation, cerebrovascular diseases, hypertension, diabetes, and hypercholesterolemia; CAMCOG: Cambridge Cognitive Examination Scale; BNT: Boston Naming Test; FUCAS: Functional Cognitive Assessment Scale; GDS: Geriatric Depression Scale; HAM-D: Hamilton Depression Scale; FRSSD: Functional Rating Scale of Symptoms of Dementia; NPI: Neuropsychiatric Inventory.

**Table 2:** Parameter estimates for predictors of major NCD diagnosis.

Parameter	B	Std. Error	Wald Chi-Square	df	Sig.	Exp(B)
(Intercept)	6.45	2.76	5.45	1	0.02	633.70
[Sex = F]	−0.66	0.23	8.33	1	0.00	0.51
[Sex = M]	0					1.00
[Cardiovascular diseases = Y]	0.15	0.28	0.28	1	0.60	1.16
[Cardiovascular diseases = N]	0					1.00
[BMI ≥ 25 = Y]	0.82	0.27	9.07	1	0.00	2.27
[BMI ≥ 25 = N]	0					1.00
[WML = Y]	−0.24	0.27	0.82	1	0.37	0.79
[WML = N]	0					1.00
[Smoking = Y]	−0.29	0.36	0.65	1	0.42	0.75
[Smoking = N]	0					1.00
[Alcohol = Y]	0.86	0.29	9.04	1	0.00	2.36
[Alcohol = N]	0					1.00
[CB = 3.00]	−0.31	0.59	0.28	1	0.59	0.73
[CB = 2.00]	−0.28	0.31	0.82	1	0.36	0.75
[CB = 1.00]	−0.16	0.28	0.33	1	0.57	0.85
[CB = 0.00]	0					1.00
CAMCOG—praxis	−0.12	0.05	6.40	1	0.01	0.89
GDS	−0.14	0.06	4.80	1	0.03	0.87
Age	−0.01	0.02	0.47	1	0.49	0.99
Education	−0.04	0.03	1.45	1	0.23	0.96
Duration	0.00	0.11	0.00	1	1.00	1.00
CAMCOG—orientation	0.04	0.17	0.04	1	0.83	1.04
CAMCOG—understanding	0.12	0.17	0.49	1	0.49	1.12
CAMCOG—language	−0.09	0.05	2.67	1	0.10	0.92
CAMCOG—memory	−0.09	0.06	2.20	1	0.14	0.92
CAMCOG—perception	0.00	0.05	0.00	1	0.97	1.00
CAMCOG—time perception	0.12	0.08	1.91	1	0.17	1.12
FUCAS	−0.02	0.02	2.27	1	0.13	0.98
BNT	0.01	0.02	0.16	1	0.69	1.01
BNT—time completion	0.00	0.00	0.01	1	0.94	1.00
NPI	−0.06	0.06	0.82	1	0.36	0.95
FRSSD	0.07	0.05	2.16	1	0.14	1.07
HAM-D	0.08	0.05	2.47	1	0.12	1.08

Notes. M: Male; F: Female; Y: Yes; N: No; BMI: Body mass index; WML: White matter lesions; CB: Cerebrovascular burden; CB-0: No cerebrovascular factor among atrial fibrillation, cerebrovascular diseases, hypertension, diabetes, and hypercholesterolemia; CB-1: One cerebrovascular factor among atrial fibrillation, cerebrovascular diseases, hypertension, diabetes, and hypercholesterolemia; CB-2: Two cerebrovascular factors among atrial fibrillation, cerebrovascular diseases, hypertension, diabetes, and hypercholesterolemia; CB-3: Three cerebrovascular factors among atrial fibrillation, cerebrovascular diseases, hypertension, diabetes, and hypercholesterolemia; CAMCOG: Cambridge Cognitive Examination Scale; GDS: Geriatric Depression Scale; FUCAS: Functional Cognitive Assessment Scale; BNT: Boston Naming Test; NPI: Neuropsychiatric Inventory; FRSSD: Functional Rating Scale of Symptoms of Dementia; HAM-D: Hamilton Depression Scale.

## Data Availability

Due to institutional policies, research data are available upon reasonable request.
